# Analysis of the synergistic antifungal activity of everolimus and antifungal drugs against dematiaceous fungi

**DOI:** 10.3389/fcimb.2023.1131416

**Published:** 2023-02-22

**Authors:** Lulu An, Gengpei Jia, Jingwen Tan, Lianjuan Yang, Yuemei Wang, Lei Li

**Affiliations:** ^1^ Department of Medical Mycology, Shanghai Skin Disease Hospital, Tongji University School of Medicine, Shanghai, China; ^2^ Department of General Medicine, Jingzhou Hospital Affiliated to Yangtze University, Jingzhou, Hubei, China; ^3^ Department of Clinical Laboratory, Shibei Hospital, Shanghai, China

**Keywords:** everolimus, synergistic effect, chromoblastomycosis, antifungal, dematiaceous fungi

## Abstract

**Introduction:**

Chromoblastomycosis (CBM) is a form of chronic mycosis that affects the skin and mucous membranes and is caused by species of dematiaceous fungi including Exophiala spp., Phialophora spp., and Fonsecaea spp. The persistence of this disease and limitations associated with single-drug treatment have complicated efforts to adequately manage this condition.

**Methods:**

In this study, a microdilution assay was used to explore the synergistic antifungal activity of everolimus (EVL) in combination with itraconazole (ITC), voriconazole (VRC), posaconazole (POS), and amphotericin B (AMB) against a range of clinical dematiaceous fungal isolates.

**Results:**

These analyses revealed that the EVL+POS and EVL+ITC exhibited superior in vitro synergistic efficacy, respectively inhibiting the growth of 64% (14/22) and 59% (13/22) of tested strains. In contrast, the growth of just 9% (2/22) of tested strains was inhibited by a combination of EVL+AMB, and no synergistic efficacy was observed for the combination of EVL+VRC.

**Discussion:**

Overall, these findings indicate that EVL holds promise as a novel drug that can be synergistically combined with extant antifungal drugs to improve their efficacy, thereby aiding in the treatment of CBM.

## 1 Introduction

Rates of dematiaceous melanized fungi-related infections have risen in recent years, including diseases such as chromoblastomycosis (CBM), phaeohyphomycosis (PHM), and mycetoma that most commonly develop in subtropical and tropical regions (Lupi et al., 2005; Revankar and Sutton, 2010). CBM, which is primarily an occupational fungal disease, exhibits a global burden that is similar to or greater than that associated with mycetoma. Given its global distribution, disproportional impact on impoverished individuals, and refractory nature, CBM represents an important but often overlooked disease (Hotez et al., 2020; Santos et al., 2021). Fungi responsible for CBM infections include members of the *Exophiala* (*E. moniliae*)*, Phialophora* (*P. verrucosa, P. chinenis, P. macroaphora, P. americana*), and *Fonsecaea* (*F. pedrosoi, F. monophora*) genera, among others (Najafzadeh et al., 2011; Li et al., 2017; Ahmed et al., 2021). While infections by these fungi generally begin in the skin, if they are not detected in the early stages of disease they can invade deep into the underlying tissues (Seas and Legua, 2022). CBM is a form of chronic mycosis, and the patients undergoing long-term treatment may develop acquired resistance to antifungal drugs, contributing to a high risk of recurrent disease (Sousa et al., 2022). Treatment with a combination of antifungal drugs is common in individuals with severe invasive infections, but there have been few studies of combination drug therapy specifically focused on dematiaceous fungi. As such, it is vital that researchers specifically test the *in vitro* activity of specific therapeutic combinations against dematiaceous fungi in an effort to explore approaches to overcoming the limitations associated with current antifungal treatment strategies.

The target of rapamycin (TOR) signaling pathway is best studied as a therapeutic target in cancer, but given its central role in the regulation of cell cycle progression, stress responsivity, and protein synthesis, it may also represent a viable antifungal target (Fingar and Blenis, 2004). The TOR inhibitor sirolimus (rapamycin) was recently shown to disrupt the survival, morphogenesis, and stress responses of *Candida* and *Aspergillus* species while decreasing *Candida* azole tolerance (Kumari et al., 2022). Combining sirolimus with itraconazole (ITC), posaconazole (POS), and amphotericin B (AMB) also reportedly yields synergistic antifungal efficacy against *Mucorales* (Dannaoui et al., 2009). However, the robust immunosuppressive effects of sirolimus on humans limit its value as an antifungal drug.

The TOR kinase inhibitor everolimus (EVL) exhibits a high level of potency and oral bioactivity such that it has received FDA approval as a prophylactic therapy for a range of malignant tumor types with minimal toxicity (Yao et al., 2016). As therapeutic targets in tumors and fungi are very similar, EVL has been explored as a treatment for various fungal infections albeit not as a treatment for dematiaceous fungi (Jiang et al., 2022). This study was thus designed to evaluate the combined effects of EVL with AMB and a range of azole antifungal drugs in an effort to determine whether these drugs would exhibit synergistic activity against a range of clinical *Exophiala* spp.*, Phialophora* spp., and *Fonsecaea* spp. isolates, which are the primary fungi responsible for CBM cases.

## 2 Results

### 2.1 Analysis of the single-agent *in vitro* activity of tested drugs

Initially, the antifungal activity of the tested drugs of interest against a range of dematiaceous fungi was tested using a checkerboard broth microdilution approach. MIC value ranges for single-agent treatment with EVL, ITC, VRC, POS, and AMB were > 16 μg/mL, 0.063-1 μg/mL, 0.031-2 μg/mL, 0.031-1 μg/mL, and 0.125-4 μg/mL, respectively (**Table 1**).

**Table 1:** Combination activity of EVL and antifungal drugs against dematiaceous fungi isolates.

No.	Species	MICs (μg/mL)^a^								
		Agents alone^b^					Combination^c^			
		EVL	ITC	VRC	POS	AMB	EVL/ITC	EVL/VRC	EVL/POS	EVL/AMB
00017	*E. moniliae*	>16	0.5	0.063	0.5	0.5	2/0.125(S)	0.25/0.063(I)	2/0.125(S)	2/0.125(S)
215-2133	*P. verrucosa*	>16	0.125	0.063	0.25	4	1/0.063(I)	0.25/0.031(I)	2/0.063(S)	0.25/4(I)
00104	*P. chinenis*	>16	1	0.25	0.5	2	2/0.031(I)	16/0.125(I)	2/0.031(S)	0.25/2(I)
00106	*P. macroaphora*	>16	0.063	0.063	0.063	0.5	0.25/<0.031(S)	0.25/0.031(I)	0.5/<0.031(I)	0.25/0.5(I)
0047		>16	0.125	0.063	0.125	2	1/0.031(S)	0.25/0.031(I)	1/0.031(S)	0.25/2(I)
01236		>16	0.125	0.125	0.25	2	2/0.031(S)	2/0.063(I)	2/0.063(S)	2/1(I)
CBS273.37		>16	0.25	0.031	0.125	0.5	2/0.063(S)	0.25/0.031(I)	1/0.031(S)	0.25/0.5(I)
00107	*P. americana*	>16	1	0.25	1	0.125	4/0.125(S)	0.25/0.25(I)	8/0.031(S)	0.25/0.125(I)
00109		>16	0.125	0.25	1	1	2/0.125(I)	0.25/0.25(I)	2/0.125(S)	0.25/1(I)
00121		>16	0.063	0.125	1	0.25	0.25/0.063(I)	0.25/0.125(I)	4/0.25(S)	0.25/0.25(I)
CBS225.97		>16	1	0.5	0.25	0.5	2/0.5(I)	0.25/0.5(I)	2/0.125(I)	0.25/0.5(I)
CBS281.35		>16	0.5	0.25	0.125	1	2/0.125(S)	0.25/0.25(I)	1/0.063(I)	2/0.25(S)
CBS400.67		>16	0.5	2	0.25	4	0.25/0.5(I)	0.25/2(I)	4/0.125(I)	0.25/4(I)
CBS840.69		>16	1	0.063	0.25	2	4/0.125(S)	2/0.063(I)	0.25/0.25(I)	0.25/2(I)
0009		>16	0.25	0.125	0.5	0.25	2/0.063(S)	8/0.063(I)	1/0.063(S)	0.25/0.25(I)
JZ202205		>16	0.125	0.063	0.25	2	0.5/0.063(I)	0.25/0.063(I)	1/0.031(S)	0.25/2(I)
07631	*F. monophora*	>16	0.25	0.125	0.25	2	8/0.063(S)	0.25/0.125(I)	0.5/0.125(I)	0.25/2(I)
07632		>16	0.25	0.063	0.063	2	1/0.063(S)	8/0.031(I)	0.25/<0.031(S)	0.25/2(I)
07633		>16	0.25	0.25	0.25	2	1/0.063(S)	0.25/0.25(I)	4/0.125(S)	0.25/2(I)
07671	*F. pedrosoi*	>16	0.125	0.063	0.063	1	16/0.031(I)	8/0.031(I)	0.25/0.031(I)	0.25/1(I)
07690		>16	0.125	0.25	0.031	1	0.25/0.125(I)	0.25/0.25(I)	0.25/<0.031(I)	0.25/1(I)
1000		>16	1	0.125	0.5	1	4/0.25(S)	0.25/0.125(I)	4/0.125(S)	0.25/1(I)

a Minimum inhibitory concentration (MIC) values are the concentrations that inhibited 100% of fungal growth.

b EVL, ITC, VRC, POS, AMB respectively correspond to everolimus, voriconazole, itraconazole, posaconazole, amphotericin B.

c Fractional inhibitory concentration index (FICI) results are provided in parentheses. S, synergism (FICI ≤ 0.5); I, indifference (0.5 < FICI < 4).

### 2.2 Analysis of the synergistic effects of combinations of EVL and antifungal agents

The combination of EVL and ITC was associated with reductions in the MIC values for these compounds to 0.25-16 μg/mL and < 0.031-0.5 μg/mL, with evidence of synergistic efficacy against 100% of *E. moniliae*, *P. macroaphora*, and *F. monophora* strains, 44% of *P. americana* strains, and 33% of *F. pedrosoi* strains included in this analysis (**Tables 1** and **2**).

**Table 2:** *In vitro* drug interactions summary.

Species (n)	n (%) of isolates showing S (synergism) for the combination			
	EVL/ITC	EVL/VRC	EVL/POS	EVL/AMB
*E. moniliae* (1)	1 (100%)	0 (0%)	1 (100%)	1 (100%)
*P. verrucosa* (1)	0 (0%)	0 (0%)	1 (100%)	0 (0%)
*P. chinenis* (1)	0 (0%)	0 (0%)	1 (100%)	0 (0%)
*P. macroaphora* (4)	4 (100%)	0 (0%)	3 (75%)	0 (0%)
*P. americana* (9)	4 (44%)	0 (0%)	5 (56%)	1 (11%)
*F. monophora* (3)	3 (100%)	0 (0%)	2 (67%)	0 (0%)
*F. pedrosoi* (3)	1 (33%)	0 (0%)	1 (33%)	0 (0%)
Total (22)	13 (59%)	0 (0%)	14 (64%)	2 (9%)

ITC, itraconazole; VRC, voriconazole; POS, posaconazole; AMB, amphotericin B; EVL, Everolimus.

The combination of EVL and POS was associated with reductions in the MIC values for these compounds to 0.25-8 μg/mL and < 0.031-0.25 μg/mL, with evidence of strong synergistic efficacy against 100% of *E. moniliae*, *P. verrucosa*, *P. chinenis* strains, 75% of *P. macroaphora* strains, 67% of *F. monophora* strains, 56% of *P. americana* strains and 33% of *F. pedrosoi* strains included in this analysis.

The combination of EVL and AMB was associated with reductions in the MIC values for these compounds to 0.25-2 μg/mL and 0.125-4 μg/mL, with evidence of synergistic efficacy against 100% of *E. moniliae* strains, although it exhibited poor synergistic activity just against 11% of *P. americana* strains.

The combination of EVL and VRC was associated with reductions in the MIC values for these compounds to 0.25-16 μg/mL and 0.031-2 μg/mL, but this combination did not exhibit synergistic efficacy against any tested *E. moniliae*, *Phialophora* spp., or *Fonsecaea* spp.

No antagonistic interactions between EVL and any of these antifungal agents were detected through this *in vitro* experimental approach.

## 3 Discussion

Here, we describe the synergistic treatment of EVL with azoles and AMB against CBM, which is characterized by sclerotic cells corresponding to the parasitic stage of the fungus together with a pronounced pyogenic and granulomatous tissue reaction in the infected host (Queiroz-Telles et al., 2009; Queiroz-Telles et al., 2017). No standard drug regimens or clinical procedures for this disease currently exist, hampering efforts to cure affected patients. A range of therapeutic strategies have been proposed to date including cryotherapy, surgery, and chemotherapy together with antifungal treatment, but the disease is often refractory to many treatments such that cure rates range from 15-80% (Heidrich et al., 2021). Therapeutic efficacy is largely tied to disease severity, with relapses being common and often disabling.

The antifungal agents most frequently used to treat CBM include ITC and terbinafine (TBF). *F. pedrosoi* strains generally exhibit good susceptibility to ITC, whereas they are resistant to AMB, 5-flucytosine, and fluconazole. However, concerns have been expressed regarding the emergence of ITC resistance in CBM patients that undergo prolonged treatment using this drug. While combinations of drugs can achieve good clinical outcomes and enhance the efficacy of individual agents, there have been few studies to date on the impact of different combination drug regimens on melanized fungi. In one prior study testing the effects of antifungal drugs (AMB, ITC, and TBF) against CBM-causing fungi including *P. verrucosa*, *C. carrionii*, and *F. pedrosoi* isolates (Yu et al., 2008), no synergistic or antagonistic effects were observed when combining AMB with TBF or ITC for any of the tested fungal target strains, although synergy between TBF and ITC was observed for one *C. carrionii* isolate. While combining antifungal treatments represents a promising approach to treating severe invasive mycoses, there is thus currently a lack of sufficient information regarding the efficacy of different antifungal agents when applied in combination, emphasizing a need to study antifungal drug synergies in greater detail (Daboit et al., 2014).

A range of targets for novel anticancer drugs exhibit homologous proteins in fungi that are involved in key cell signaling pathways (Bastidas et al., 2008; Chen et al., 2011). As such, efforts to investigate these targets and inhibitors thereof may highlight new avenues for antifungal treatment. TOR is a highly conserved serine/threonine kinase that regulates metabolic activity and growth in eukaryotic cells (Blenis, 2017). Mammalian TOR (mTOR) dysregulation is believed to play important roles in autoimmunity, cancer, cardiovascular disease, metabolic disorders, and fungal virulence and pathogenicity (Yu et al., 2014; Mossmann et al., 2018; So et al., 2019). Efforts to target TOR signaling may thus be of value for the treatment of both cancer and fungal infections. In prior studies, rapamycin was shown to exert antifungal effects and to reverse *C. albicans* drug resistance through the suppression of TOR signaling, although its intrinsic toxicity greatly limited its antifungal application (Tong et al., 2021).

EVL is a less toxic rapamycin analog that exhibits excellent oral bioavailability and has been used to treat a range of cancers including breast, lung, and pancreatic tumors (Babiker et al., 2019). In precious several studies, the EVL-induced cell survival rate in HaCat, Caki-1, and HepG2 cells were greater than 70% at 30 μM (≈28.75 μg/mL), especially up to 100 μM in HepG2 cells (Yamamoto et al., 2013; Navarro-Villarán et al., 2016). Recent studies have also demonstrated the activity of EVL against *Aspergillus*, *Scedosporium*, and *Lomentospora* species (Wang et al., 2022). However, there have not been any published studies assessing the effects of EVL against dematiaceous fungi. Given the relevance of *Exophiala, Phialophora*, and *Fonsecaea* species as important pathogens that can cause CBM, this study was performed to examine the combined therapeutic effects of EVL and existing antifungal drugs against these fungi.

Here, the antifungal effects of EVL alone and in combination with different antifungal drugs were tested. In total, 22 clinical *Exophiala*, *Phialophora*, and *Fonsecaea* isolates were used for this testing effort. These analyses ultimately revealed that EVL exhibited synergistic efficacy when combined with AMB or azoles, resulting in significant reductions in the MIC values for these antifungal agents. When combined with EVL, the MIC range for ITC declined from 0.063-1 μg/mL to < 0.031-0.5 μg/mL, with a total synergy rate of 59%. Similarly, combining POS with EVL resulted in the best synergistic efficacy, with decreases in MIC ranges from 0.063-1 μg/mL to < 0.031-0.25 μg/mL and a total synergy rate of 64%. In contrast, combining EVL and AMB yielded a relatively weak synergy rate (9%), while the combination of EVL and VRC failed to exhibit synergistic efficiency, likely as the analyzed strains were sensitive to single-agent VRC.

Given that the long-term drug treatment of CBM patients can contribute to the emergence of therapeutic resistance, this study was developed to examine the synergistic effects of EVL in combination with other antifungal drugs *in vitro* against a range of *Exophiala*, *Phialophora*, and *Fonsecaea* isolates. These results demonstrate that combining EVL with azoles and AMB represents a promising approach to treating diseases caused by these fungi, although additional work will be necessary to clarify the mechanisms underlying this synergistic activity and to test whether these results translate to *in vivo* efficacy.

## 4 Materials and methods

### 4.1 Reagents

EVL (RAD001, 99.69%) was obtained from Selleck Chemicals LLC (TX, USA). ITC and AMB were from Macklin (Shanghai, China). VRC and POS were from Solarbio Biotechnology (Beijing, China). RPMI-1640 was from Gen-view Scientific Inc. (FL, USA). All reagents were prepared as per Clinical and Laboratory Standards Institute M38-A2 guidelines (CLSI, 2008).

### 4.2 Fungal isolates

For this study, 22 different dematiaceous fungi isolates were utilized including one *E. moniliae*, one *P. verrucosa*, one *P. chinenis*, four *P. macroaphora*, nine *P. americana*, three *F. monophora*, and three *F. pedrosoi* strains, all of which were clinical isolates. *Candida parapsilosis* ATCC 22019 and *Aspergillus flavus* ATCC 204304 strains were used for the purposes of quality control. Strain identities were confirmed through a combination of morphological identification and ITS sequencing. All specimens were deposited in the Department of Dermatology of Jingzhou Central Hospital.

### 4.3 Inoculum preparation

After incubation for 4 days on potato dextrose agar (PDA) at 28°C, conidia were prepared from all strains at 1-5 × 10^6^ conidia/mL in 0.9% sterile saline followed by 100-fold dilution using RPMI-1640 to a final concentration of 1-5 × 10^4^ conidia/mL.

### 4.4 Checkerboard microdilution method

The minimum inhibitory concentration (MIC) values of EVL alone or in combination with ITC, VRC, POS, or AMB against various dematiaceous fungi were assessed as per the guidelines established by the Clinical and Laboratory Standards Institute (CLSI) M38-A2. The working concentrations for the tested drugs were 0.06-4 μg/mL for ITC, VRC, POS, and AMB, and 0.25-16 μg/mL for EVL. Briefly, 50 μl of serially diluted EVL solutions were added to horizontal rows of a 96-well plate containing the conidia suspension prepared above (100 μl/well), followed by the addition of 50 μl of serially diluted ITC, VRC, POS, or AMB in the vertical columns of this plate. Plates were then incubated for 4 days at 28°C, with MIC values then being established based on the minimum drug concentration necessary to suppress 100% of fungal growth relative to control conditions.

Interactions between EVL and specific antifungal drugs were assessed based on the fractional inhibitory concentration index (FICI) as follows: FICI = (MIC_Ac_/MIC_Aa_) + (MIC_Bc_/MIC_Ba_), where MIC_Ac_ and MIC_Bc_ respectively correspond to test drug combinations, and MIC_Aa_ and MIC_Ba_ correspond to the MIC values for drugs A and B when used as single-agent treatments. A FICI ≤ 0.5 was indicative of synergism, while 0.5 < FICI < 4 indicated no interaction or indifference, and FICI ≥ 4 indicated antagonism. All experiments were independently repeated in triplicate.

## Data availability statement

The original contributions presented in the study are included in the article/supplementary material. Further inquiries can be directed to the corresponding author.

## Author contributions

LA and GJ carried out the whole experiment. JT and LY collected and analyzes the data of experiment. LL and LA designed the whole experiment, and wrote the manuscript. YW revised the manuscript critically for important content. All authors contributed to the article and approved the submitted version.
